# Single and Associated Effects of Drought and Heat Stresses on Physiological, Biochemical and Antioxidant Machinery of Four Eggplant Cultivars

**DOI:** 10.3390/plants11182404

**Published:** 2022-09-15

**Authors:** Sami Hannachi, Angelo Signore, Mohd Adnan, Lassaad Mechi

**Affiliations:** 1 Department of Biology, College of Science, University of Hail, P.O. Box 2440, Ha’il 81451, Saudi Arabia; 2 Department of Plants and Crops, Faculty of Bioscience Engineering, Ghent University, Coupure Links, 653, 9000 Ghent, Belgium; 3 Department of Agricultural and Environmental Science, University of Bari Aldo Moro, Via Amendola 165/A, 70126 Bari, Italy; 4 Department of Chemistry, College of Science, University of Hail, P.O. Box 2440, Ha’il 81451, Saudi Arabia

**Keywords:** ROS, MDA, chlorophyll fluorescence, antioxidants, electrolyte leakage, mineral uptake, photosynthesis, *Solanum melongena* L.

## Abstract

The impact of heat and drought stresses, either individually or combined, on physiological and biochemical parameters of four eggplant varieties (*Solanum melongena* L.) was investigated. The results showed that associated stress generated the highest increment in proline content, MDA concentration, and H_2_O_2_ accumulation and generated the lowest increment in RWC. In addition, ‘Bonica’ and ‘Galine’ exhibited higher starch accumulation and lower electrolyte leakage (EL) under combined stress. Moreover, drought and heat stresses applied individually contributed to a substantial decline in Chl*a*, Chl*b*, total Chl, Chl*a/b*, and carotenoids (*p* > 0.05) in ‘Adriatica’ and ‘Black Beauty’. The decreasing level of pigments was more substantial under associated drought and heat stresses. The simultaneous application of drought and heat stresses reduced PSII efficiency (F_v_/F_m_), quantum yield (ΦPSII), and photochemical efficiency (q_p_) and boosted non-photochemical quenching (NPQ) levels. However, the change recorded in the chlorophyll fluorescence parameters was less pronounced in ‘Bonica’ and ‘Galine’. In addition, the gas exchange parameters, transpiration rate (E), CO_2_ assimilation rate (A), and net photosynthesis (Pn) were decreased in all varieties under all stress conditions. However, the reduction was more pronounced in ‘Adriatica’ and ‘Black Beauty’. Under associated stress, antioxidant enzymes, SOD, APX, CAT, and GR exhibited a significant increment in all eggplant cultivars. However, the rising was more elevated in ‘Bonica’ and ‘Galine’ (higher than threefold increase) than in ‘Adriatica’ and ‘Black Beauty’ (less than twofold increase). Furthermore, ‘Bonica’ and ‘Galine’ displayed higher non-enzyme scavenging activity (AsA and GSH) compared to ‘Adriatica’ and ‘Black Beauty’ under associated stress. Under stressful conditions, nutrient uptake was affected in all eggplant cultivars; however, the root, stem, and leaf N, P, and K contents, in ‘Adriatica’ and ‘Black Beauty’ were lower than in ‘Bonica’ and ‘Galine’, thereby showing less capacity in accumulating nutrients. The coexistence of drought and heat stresses caused more damage on eggplant varieties than the single appearance of drought or heat stress separately. ‘Bonica’ and ‘Galine’ showed better distinguished performance compared to ‘Adriatica’ and ‘Black Beauty’. The superiority of ‘Bonica’ and ‘Galine’ in terms of tolerance to heat and drought stresses was induced by more effective antioxidant scavenging potential, enhanced osmolyte piling-up, and prominent ability in keeping higher photosynthetic efficiency and nutrient equilibrium compared with ‘Adriatica’ and ‘Black Beauty’.

## 1. Introduction

Under field conditions, crops face a combined effect of several abiotic stresses, which induce simultaneous effects on growth and production during the plant life cycle [1,2,3].

Traditionally, most reports emphasize the evaluation of each abiotic stress response separately [4,5,6]. However, the co-occurrence of abiotic stresses under field conditions is unavoidable and is more drastic than single-factor impacts. Consequently, a new approach based on the study of the overlapping effects of abiotic stresses seems to be of immense importance to analyze plants’ responses under environmental conditions [2,3]. High temperature and drought are the most frequent combined threats to crops productivity [2,3]. The optimum temperature for eggplant is species-dependent [7] and is between 22 and 30 °C [8,9,10]. Nonetheless, the reproductive phases of the crop plants are more susceptible to the co-occurrence of drought and heat stresses than the vegetative phases [1,11]. The reduction of precipitation in open fields generates the occurrence and the intensification of drought stress which, in association with higher temperature, contributes to an increase in plant tissue temperature [12]. Under heat stress, the rapid water loss from the soil surface and the plant ultimately leads to occurrence of drought stress conditions [3]. Moreover, heat stress might appear in condition of low water availability in order to compensate for evaporation loss [13].

Both stress factors decrease photosynthetic efficiency, affect oxidative metabolism, generate membrane instability [14,15], alter stomatal conductance, and reduce leaf area and water-use efficiency in several crops [16,17,18]. Heat stress increases electrolyte leakage, as a measure of cell membrane damage, and intensifies proline accumulation [19]. Heat and drought stresses contribute to a decrease in nutrient uptake and a decline in plant photosynthesis [20].

In eggplant crops, heat stress hampers plant growth, decreases productivity, and affect fruit quality [21].

Previous reports [7,20] showed that heat and drought stresses induced comparable plant behaviors. Yet, the effect of the two associated factors was more drastic than the impact of each stress applied separately. In this context, there is a growing number of arguments showing that plants develop a complex set of reactions to counteract the combined effect of drought and heat stresses [3]. The synthesis of compatible solutes (sugars, proline) and the adoption of enzymatic (catalase, CAT; peroxidase, POD, ascorbate; peroxidase, APX) and non-enzymatic mechanisms (reduced glutathione, GSH) constitute efficient strategies that plants use to adapt to stress conditions [22,23,24]. Under abiotic stress combination, a whole set of plant responses occurs, ranging from variation in major stress-signaling molecules, such as free radicals [25] and plant hormones [26], up to accelerated death via inhibition of stem hydraulic conductivity [27].

Plant growth-promoting rhizobacteria (PGPRs) is one of the strategies adopted by plants to overcome various abiotic stresses, promote plant growth, and effectively improve crop productivity [28,29,30,31,32].

PGPRs include many beneficial bacteria that increase plant abiotic stress tolerance by reducing the ethylene level via hydrolyzing 1-aminocyclopropane-1-carboxylic acid (ACC) into ammonia and α-ketobutyrate using the ACC deaminase [29,32,33,34]. ACC is the direct precursor of the hormone ethylene in plants [35]. In addition, indoleacetic acid (IAA) has the capacity to improve cell division and elongation, thus promoting root growth and architecture [36,37]. Eggplant (*Solanum melongena* L.), a thermophilic plant, is a well-known vegetable cultivated worldwide. Global warming engendered an increase in temperature ranging from 38 to 45 °C, mostly in semiarid areas where eggplant is mainly cultivated, which can severely alter eggplant growth, flower formation, fruit quality, and yield [38,39]. It has been reported that environmental stresses including drought, cold, and heat reduced the potential yield of major crops by more than 50% [8]. As a result of ongoing climatic change, it is expected that several crops, including eggplant, are likely to be more affected by various kind of stresses. Despite the fact that drought and heat stresses frequently coexist, most previous studies focus on the impacts of heat or drought stress separately [1]. To our knowledge, no studies have yet been conducted regarding the associated effects of drought and heat stresses on agronomical, physiological, and biochemical parameters of eggplant. This study focuses not only on characterization of the impact of individual and associated drought and heat stresses in four eggplant varieties by assessing the agronomical, physiological, and biochemical performances, but also focuses on the evaluation of eggplant tolerance or susceptibility to individual and combined drought and heat stresses.

## 2. Results

### 2.1. Growth and Yield Evaluation

All treatments (drought, heat, and combined drought and heat) altered the growth and yield parameters (height, fresh and dry weight, fruit number, and fruit weight) in all varieties. The drought and heat stress conditions, alone or in combination, significantly decreased the plant height, fresh weight, dry weight, fruit number, and fruit weight in ‘Adriatica’, ‘Black Beauty’, ‘Bonica’, and ‘Galine’, compared to their respective controls (Table 1 and Table 2). When considered separately, the impact of drought stress was more pronounced for all parameters in all varieties in comparison with heat stress. Under all drought, heat, and combined drought and heat conditions, the growth and yield parameters of ‘Bonica’ and ‘Galine’ were higher than those of ‘Adriatica’ and ‘Black Beauty’ (Table 1 and Table 2).

### 2.2. Individual and Combined Drought and Heat Stress-Induced Changes in Pigments, Chlorophyll Fluorescence, and Gas Exchange Parameters

All studied stressors hardly affected Chl*a*, Chl*b*, total Chl, Chl*a/b*, and carotenoids (*p* > 0.05) in ‘Bonica’ and ‘Galine’; however, a drastic reduction was noticed in all pigments under all stress types in ‘Adriatica’ and ‘Black Beauty’ (Table 3). Moreover, the impact of heat stress was more pronounced than drought in all leaf pigments for all cultivars (Table 3).

All examined stresses significantly decreased levels of chlorophyll a (Chl*a*), chlorophyll b (Chl*b*), Chl*a*+*b*, Chl*a*/*b*, and carotenoids in ‘Adriatica’ and ‘Black Beauty’, showing their susceptibility under stressful environments. However, the same parameters remained quite stable in ‘Bonica’ and ‘Galine’, indicating their higher tolerance under stress conditions (Table 3).

**Figure 1:**
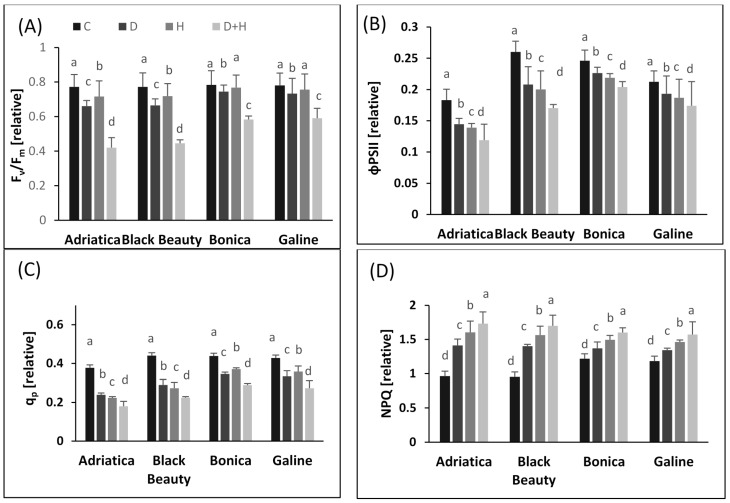
Effect of single and associated stresses on chlorophyll fluorescence parameters in four eggplant varieties. (**A**): PSII efficiency (F_v_/F_m_); (**B**): PSII quantum yield (ΦPSII); (**C**): photochemical efficiency (q_p_); (**D**): non-photochemical quenching (NPQ). Data represents means ± SE (*n* = 5). Significant dissimilarities are shown by different lower-case letters between treatments (*p* ≤ 0.05) based on Tukey’s test. (C, control; H, heat stress; D, drought stress; H + D, heat + drought stresses).

In comparison to control, all chlorophyll fluorescence parameters were decreased by all the stress treatments in all varieties (Figure 1). However, the reduction was stronger in ‘Adriatica’ and ‘Black Beauty’ than in ‘Bonica’ and ‘Galine’. Under drought stress alone, PSII efficiency (F_v_/F_m_) decreased in ‘Adriatica’, ‘Black Beauty’, ‘Bonica’, and ‘Galine’ by 14.5%, 13.9%, 5%, and 6%, respectively (Figure 1A). Further, under heat stress alone, PSII efficiency (F_v_/F_m_) was reduced by 7.2%, 6.9%, 2%, and 3% in ‘Adriatica’, ‘Black Beauty’, ‘Bonica’, and ‘Galine’, respectively (Figure 1A). However, the combined stress had a higher impact on F_v_/F_m_, which was diminished by 45.5%, 42.3%, 25.6%, and 24.3% in ‘Adriatica’, ‘Black Beauty’, ‘Bonica’, and ‘Galine’, respectively (Figure 1A). The associated effect of heat and drought stresses resulted in a larger decline in PSII quantum yield (ΦPSII) (Figure 1B) and photochemical efficiency (q_p_) (Figure 1C) in all varieties compared to individual stresses. However, the decrease was harsher in ‘Adriatica’ and ‘Black Beauty’ than in ‘Bonica’ and ‘Galine’ (Figure 1B,C). Under drought stress alone, the ΦPSII was lowered by about 21%, 20%, 9%, and 8% in ‘Adriatica’, ‘Black Beauty’, ‘Galine’, and ‘Bonica’, respectively (Figure 1B). Moreover, the combined stresses decreased ΦPSII by about 35%, 34.5%, 18%, and 17% in ‘Adriatica’, ‘Black Beauty’, ‘Galine’, and ‘Bonica’, respectively (Figure 1B). Furthermore, under drought stress alone, a reduction of about 36.5%, 35%, 22%, and 21% was observed respectively in ‘Adriatica’, ‘Black Beauty’, ‘Galine’, and ‘Bonica’ in the case of photochemical efficiency (q_p_) (Figure 1C). Added to that, the heat stress alone decreased the q_p_ by 41%, 38%, 16.5%, and 15.3% in ‘Adriatica’, ‘Black Beauty’, ‘Galine’, and ‘Bonica’, respectively (Figure 1C). However, the combined stresses lowered the photochemical efficiency by 52.3%, 49.2%, 36.2%, and 34.1%, respectively, in ‘Adriatica’, ‘Black Beauty’, ‘Galine’, and ‘Bonica’ (Figure 1C).

In addition, all stress conditions increased the non-photochemical quenching (NPQ) in all varieties (Figure 1D). However, the NPQ was further increased under combined stresses. Drought stress boosted the non-photochemical quenching by 47%, 46.5%, 13.5%, and 12.3% in ‘Black Beauty’, ‘Adriatica’, ‘Galine’, and ‘Bonica’, respectively (Figure 1D). Heat stress alone rose the NPQ by 66.3%, 64.2%, 23.5%, and 22.6% in ‘Adriatica’, ‘Black Beauty’, ‘Galine’, and ‘Bonica’, respectively (Figure 1D). However, the combined stresses increased the non-photochemical quenching by 79.5%, 78.2%, 32.8%, and 31.2%, respectively, in ‘Adriatica’, ‘Black Beauty’, ‘Galine’, and ‘Bonica’ (Figure 1D).

**Figure 2:**
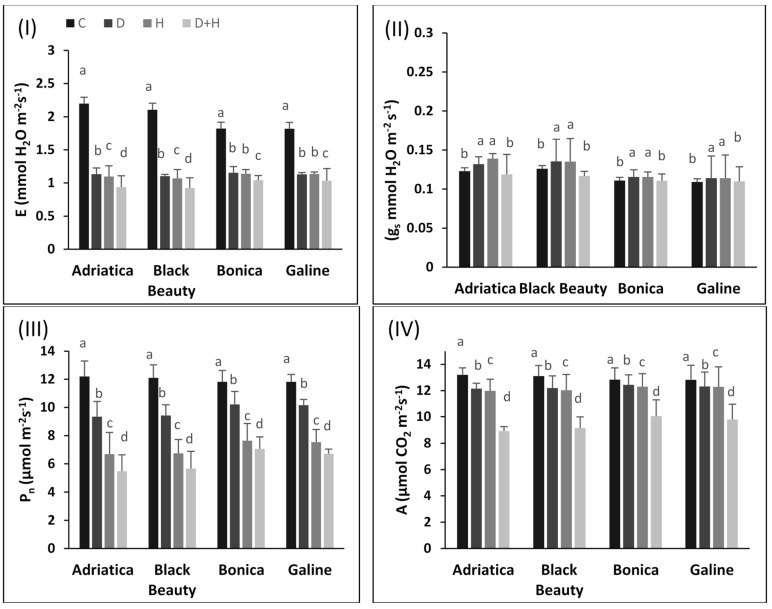
Changes in photosynthesis and gas exchange parameters under individual and combined stresses. (**I**) Transpiration rate (E), (**II**) stomatal conductance (gs), (**III**) net photosynthesis (Pn), and (**IV**) CO_2_ assimilation rate (A) in four eggplant varieties. Data presented are the means ± SE (*n* = 5). Significant dissimilarities are shown by different lower-case letters between treatments (*p* ≤ 0.05) based on Tukey’s test. (C, control; H, heat stress; D, drought stress; H + D, heat + drought stresses).

The gas exchange parameters (Figure 2I–IV), namely transpiration rate (E), stomatal conductance (gs), CO_2_ assimilation rate (A), and net photosynthesis (Pn), were declined in all varieties under all stress conditions (Figure 2I–IV). However, the decrease was more pronounced in ‘Adriatica’ and ‘Black Beauty’ than in ‘Bonica’ and ‘Galine’.

Drought stress alone decreased transpiration rate by 48.3%, 47.5%, 37.9%, and 36.6% in ‘Adriatica’, ‘Black Beauty’, ‘Galine’, and ‘Bonica’, respectively. Moreover, heat stress alone reduced transpiration rate by 50.1%, 49.1%, 38.6%, and 37.5% in ‘Adriatica’, ‘Black Beauty’, ‘Galine’, and ‘Bonica’, respectively. However, combined stresses decreased transpiration rate by 57.6%, 56.2%, 43.2%, and 42.8% in ‘Adriatica’, ‘Black Beauty’, ‘Galine’, and ‘Bonica’, respectively (Figure 2I).

The stomatal conductance diminished in all varieties under all stress conditions. However, the increase was less exacerbated in ‘Bonica’ and ‘Galine’ than in ‘Adriatica’ and ‘Black Beauty’. Drought stress alone rose the stomatal conductance by 7.3%, 7.5%, 4.2%, and 4.4% in ‘Adriatica’, ‘Black Beauty’, ‘Bonica’, and ‘Galine’, respectively. In addition, heat stress alone boosted stomatal conductance by 7.2%, 7.4%, 4.3%, and 4.5% in ‘Adriatica’, ‘Black Beauty’, ‘Bonica’, and ‘Galine’, respectively. However, the stomatal conductance was more aggravated under combined stresses in all varieties (Figure 2II).

Net photosynthesis declined by 23.5%, 22.1%, 13.6%, and 14.1% in ‘Adriatica’, ‘Black Beauty’, ‘Bonica’, and ‘Galine’, respectively, under drought stress alone. Heat stress alone reduced net photosynthesis by 45.1%, 44.2%, 35.3%, and 36.1% in ‘Adriatica’, ‘Black Beauty’, ‘Bonica’, and ‘Galine’, respectively. However, the combined stresses further decreased net photosynthesis by 55.1%, 53.2%, 43.1%, and 40.2% in ‘Adriatica’, ‘Black Beauty’, ‘Bonica’ and ‘Galine’ respectively (Figure 2III).

In the case of CO_2_ assimilation rate, the decrease was 8%, 7%, 4%, and 3% in ‘Adriatica’, ‘Black Beauty’, ‘Galine’, and ‘Bonica’, respectively, under the drought stress condition. Heat stress alone lowered CO_2_ assimilation rate by 9.2%, 8.3%, 4.2%, and 4.1% in ‘Adriatica’, ‘Black Beauty’, ‘Galine’, and ‘Bonica’, respectively. However, the combined stresses reduced CO_2_ assimilation rate by 32.5%, 30.3%, 23.6%, and 21.6% in ‘Adriatica’, ‘Black Beauty’, ‘Galine’, and ‘Bonica’, respectively (Figure 2IV).

### 2.3. Drought-, Heat-, and Combined Drought- and Heat-Induced Changes in Relative Water Content, H_2_O_2_ Content, Malonaldehyde, Electrolyte Leakage, Starch, and Proline Concentration

**Figure 3:**
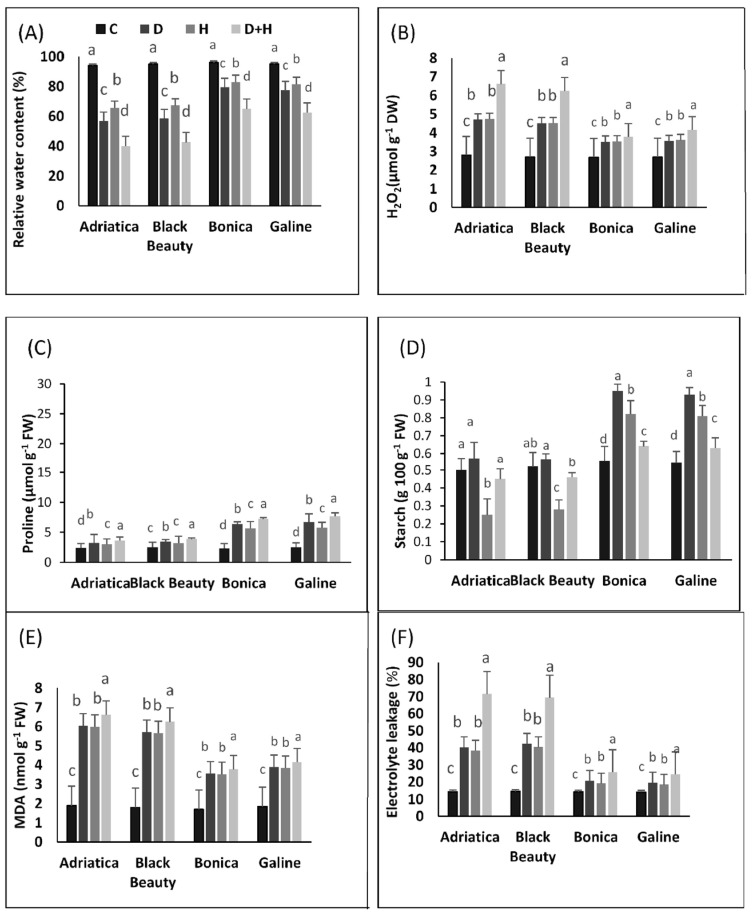
Changes in relative water content (%) (**A**), H_2_O_2_ content (μmol g^−1^ DW) (**B**), leaf proline content (μmol g^−1^ FW) (**C**), leaf starch content (g 100 g^−1^ FW) (**D**), peroxidation of leaf lipid (nmol g^−1^ FW) (**E**), and in electrolyte leakage (%) (**F**) of four eggplant cultivars under individual and combined stresses. Data presented are the means ± SE (*n* = 5). Significant dissimilarities are shown by different lower-case letters between treatments *p* ≤ 0.05 based on Tukey’s test. (C, control; H, heat stress; D, drought stress; H + D, heat + drought stresses).

The effect of the drought, heat, and their combination on relative water content, H_2_O_2_ content, malonaldehyde, electrolyte leakage, starch, and proline concentration are given in Figure 3.

In response to individual and combined stresses, RWC decreased in all eggplant cultivars. However, the reduction was more aggravated in ‘Adriatica’ and ‘Black Beauty’ than in ‘Bonica’ and ‘Galine’. Drought stress alone declined RWC by 39.6%, 38.3%, 18.5%, and 17.2% in ‘Adriatica’, ‘Black Beauty’, ‘Galine’, and ‘Bonica’, respectively. In addition, under the heat stress condition, RWC was reduced by 30.2%, 29.3%, 14.2%, and 13.6% in ‘Adriatica’, ‘Black Beauty’, ‘Galine’, and ‘Bonica’, respectively. However, combined drought and heat stresses further reduced RWC by 57.5%, 55.2%, 34.6%, and 32.3% in ‘Adriatica’, ‘Black Beauty’, ‘Galine’, and ‘Bonica’, respectively (Figure 3A).

Drought stress alone increased H_2_O_2_ levels in ‘Adriatica’, ‘Black Beauty’, ‘Galine’, and ‘Bonica’ by 68.3%, 65.1%, 31.4%, and 30.5%, respectively, when compared to control. Under heat stress alone, an elevation of 69.3%, 67.2%, 33.5%, and 31.2% was noticed in H_2_O_2_ levels in ‘Adriatica’, ‘Black Beauty’, ‘Galine’, and ‘Bonica’, respectively; however, under combined stresses, H_2_O_2_ levels showed a substantial increment of about 120.5%, 118.5%, 60.3%, and 58.2% in ‘Adriatica’, ‘Black Beauty’, ‘Galine’, and ‘Bonica’, respectively (Figure 3B).

Leaf proline rose significantly in stressed plants in all four cultivars when compared to their respective controls. However, the increase was more pronounced in ‘Adriatica’ and ‘Black Beauty’ than in ‘Bonica’ and ‘Galine’. Moreover, drought stress alone boosted proline content by 33.6%, 35.4%, 268.3%, and 277.5% in ‘Adriatica’, ‘Black Beauty’, ‘Galine’, and ‘Bonica’, respectively. In addition, heat stress alone increased proline concentration by 23.5%, 26.6%, 230.8%, and 244.8% in ‘Adriatica’, ‘Black Beauty’, ‘Galine’, and ‘Bonica’, respectively. However, combined drought and heat stresses contributed to the highest proline content increment, which reached 314.4%, 306.4%, 53.6%, and 51.2% in ‘Adriatica’, ‘Black Beauty’, ‘Galine’, and ‘Bonica’, respectively (Figure 3C).

Leaf starch content increased strongly under drought, heat, and combined stresses in ‘Bonica’ and ‘Galine’. However, under drought stress alone, no significant change was observed in ‘Adriatica’ and ‘Black Beauty’. In addition, Heat stress alone significantly decreased leaf starch reserves by 53.8% and 50% in ‘Black Beauty’ and ‘Adriatica’ (Figure 3D).

In response to individual and combined stresses, lipid peroxidation was more pronounced in ‘Adriatica’ and ‘Black Beauty’ than in ‘Bonica’ and ‘Galine’. Furthermore, the oxidative damage in terms of MDA concentration was more aggravated under combined stresses than under individual stresses. Drought stress alone rose MDA concentration by 218.5%, 217.6%, 110.02%, and 109.2% in ‘Adriatica’, ‘Black Beauty’, ‘Galine’, and ‘Bonica’. In addition, under the heat stress condition, MDA content increased by 215.6%, 214.3%, 108.5%, and 107.3% in ‘Adriatica’, ‘Black Beauty’, ‘Galine’, and ‘Bonica’, respectively. However, combined stresses elevated MDA concentration by 248.6%, 247.5%, 124.5%, and 122.7% in ‘Adriatica’, ‘Black Beauty’, ‘Galine’, and ‘Bonica’, respectively (Figure 3E).

Higher EL was noticed under combined stress conditions when compared to each stress alone in all varieties. However, the rise was more substantial in ‘Adriatica’ and ‘Black Beauty’ than in ‘Bonica’ and ‘Galine’. The imposition of combined stresses rose EL by 0.3-, 0.4-, 2.5-, and 2.7-fold in ‘Bonica’, ‘Galine’, ‘Black Beauty’, and ‘Adriatica’, respectively, compared to control plants. Drought stress alone increased EL by 0.2-, 0.25-, 2.3-, and 2.5-fold in ‘Bonica’, ‘Galine’, ‘Black Beauty’, and ‘Adriatica’, respectively, when compared to control plants. However, while heat stress alone contributed to an elevation of EL by 2-fold in ‘Adriatica’ and ‘Black Beauty’, it hardly affected the same parameter in ‘Bonica’ and ‘Galine’ compared to control plants (Figure 3F).

### 2.4. Drought-, Heat-, and Combined Drought and Heat-Induced Activity Changes in Antioxidant Enzyme Pathway

**Figure 4:**
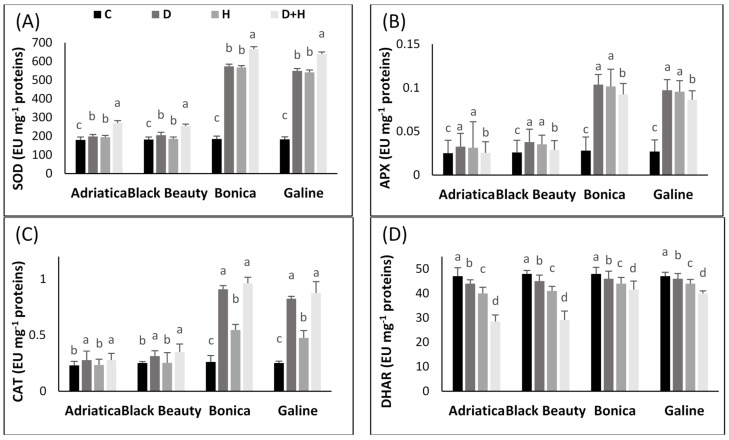
Changes in SOD (**A**), APX (**B**), CAT (**C**), and DHAR (**D**) of four eggplant cultivars under individual and combined stresses. Data presented are the means ± SE (*n* = 5). Significant dissimilarities are shown by different lower-case letters between treatments *p* ≤ 0.05 based on Tukey’s test. (C, control; H, heat stress; D, drought stress; H + D, heat + drought stresses).

The effect of stresses (either individual or combined) on the antioxidant enzyme pathway in all four eggplant cultivars is given in Figure 4.

Under combined stresses, SOD activity increased 1.4-, 1.5-, 3.5-, and 3.6-fold in ‘Black Beauty’, ‘Adriatica’, ‘Galine’, and ‘Bonica’, respectively. However, drought stress alone rose the activity of SOD by 1.1-, 1.2-, 3-, and 3.1-fold in ‘Adriatica’, ‘Black Beauty’, ‘Galine’, and ‘Bonica’, respectively. Added to that, the heat stress condition boosted SOD activity by 0.98-, 1.08-, 2.96-, and 3.07-fold in ‘Black Beauty’, ‘Adriatica’, ‘Galine’, and ‘Bonica’, respectively, when compared to control plants (Figure 4A).

The same trend showed by SOD activity was observed in APX, CAT, and GR activity under individual and combined stresses in all studied varieties. Drought stress alone rose APX activity by 1.3-, 1.45-, 3.6-, and 3.7-fold in ‘Adriatica’, ‘Black Beauty’, ‘Galine’, and ‘Bonica’, respectively, in comparison to control plants.

In addition, heat stress alone caused an increment of 1.25-, 1.36-, 3.54-, and 3.63-fold in ‘Adriatica’, ‘Black Beauty’, ‘Galine’, and ‘Bonica’, respectively. However, combined stresses increased APX activity by 1.02-, 1.15-, 3.2-, and 3.3-fold in ‘Adriatica’, ‘Black Beauty’, ‘Galine’, and ‘Bonica’, respectively, compared to control plants (Figure 4B).

Higher CAT activities were observed under combined stress conditions when compared to each stress alone in all varieties. However, the increase was more pronounced in ‘Bonica’ and ‘Galine’ than in ‘Adriatica’ and ‘Black Beauty’. The imposition of combined stresses rose CAT activity by 1.2-, 1.4-, 3.5-, and 3.7-fold in ‘Adriatica’, ‘Black Beauty’, ‘Galine’, and ‘Bonica’, respectively, compared to control plants. Drought stress alone increased CAT activity by 1.2-, 1.25-, 3.3-, and 3.5-fold in ‘Adriatica’, ‘Black Beauty’, ‘Galine’, and ‘Bonica’, respectively, in comparison to control plants. However, while heat stress alone contributed to an elevation of CAT activity by 2-fold in ‘Bonica’ and ‘Galine’, it hardly affected the same enzyme activity in ‘Adriatica’ and ‘Black Beauty’ compared to control plants (Figure 4C).

Although DHAR and MDHAR were reduced under all studied stresses in all varieties, the decline was more pronounced in ‘Adriatica’ and ‘Black Beauty’ than in ‘Bonica’ and ‘Galine’. Both MDHAR and DHAR exhibited a reduction of about 2.0-fold in ‘Adriatica’ and ‘Black Beauty’ and of about 1.2-fold in ‘Bonica’ and ‘Galine’ under combined stresses; however, neither drought nor heat stress alone had a significant effect in comparison to control (Figure 4A,D).

**Figure 5:**
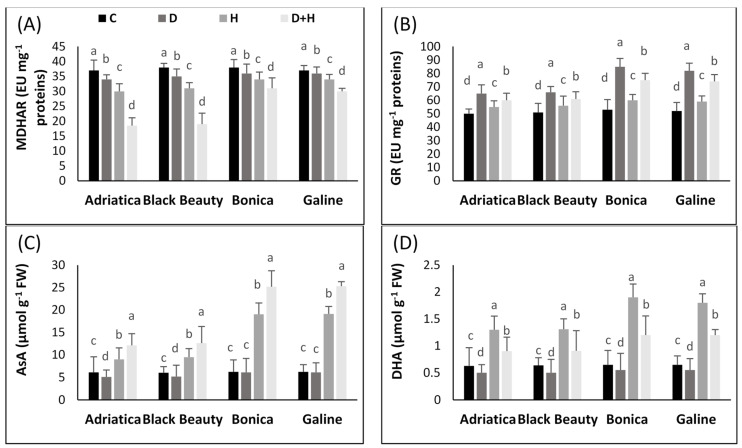
Variations in (**A**) Monodehydroascorbate reductase (MDHAR), (**B**) glutathione reductase (GR), (**C**) ascorbic acid (AsA), and (**D**) dehydroascorbate (DHA) of four eggplant cultivars under individual and combined stresses. The data are the means ± SE (*n* = 5). Significant dissimilarities are shown by different lower-case letters between treatments (*p* ≤ 0.05) based on Tukey’s test. (C, control; H, heat stress; D, drought stress; H + D, heat + drought stresses).

In addition, ‘Bonica’ and ‘Galine’ exhibited a higher increase in GR activity than ‘Adriatica’ and ‘Black Beauty’ under combined and individual stress conditions. However, heat stress alone caused a slight increment in GR activity in all varieties compared to control plants (Figure 5B).

Heat stress alone rose AsA levels by 1.6-, 1.7-, 3.2-, and 3.4-fold in ‘Adriatica’, ‘Black Beauty’, ‘Galine’, and ‘Bonica’, respectively, in comparison to control plants. However, the drought stress condition hardly affected AsA levels in all varieties (Figure 5C).

**Figure 6:**
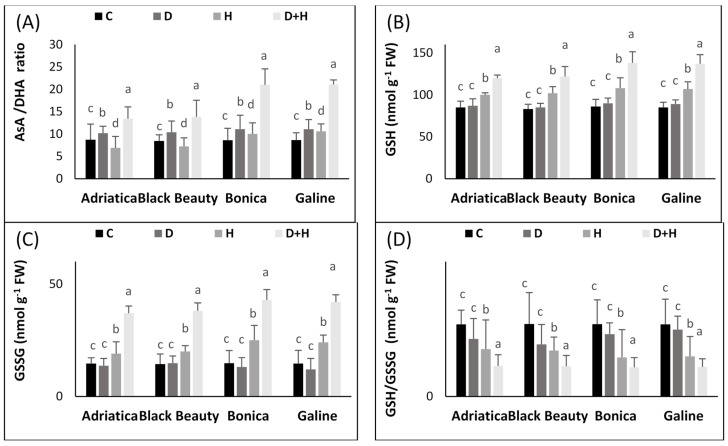
Changes in (**A**) ascorbic acid/ dehydroascorbate (AsA/DHA), (**B**) (GSH), (**C**) (GSSG), and (**D**) (GSH/GSSG) of four eggplant cultivars under individual and combined stresses. Data are the means ± SE (*n* = 5). Significant dissimilarities are shown by different lower-case letters between treatments (*p* ≤ 0.05) based on Tukey’s test. (C, control; H, heat stress; D, drought stress; H + D, heat + drought stresses).

Moreover, under drought stress alone, AsA/DHA exhibited an increase of 1.1-, 1.2-, 1.3-, and 1.3-fold in ‘Adriatica’, ‘Black Beauty’, ‘Bonica’, and ‘Galine’, respectively, when compared to control plants. Additionally, combined stresses rose the AsA/DHA ratio by 1.5-, 1.6-, 2.4-, and 2.5-fold in ‘Adriatica’, ‘Black Beauty’, ‘Galine’, and ‘Bonica’, respectively, in comparison to control plants (Figure 6A).

Under heat and combined stresses, all eggplant cultivars showed higher GSH and GSSG enhancement. However, GSH and GSSG accumulation were more important in ‘Bonica’ and ‘Galine’ than in ‘Adriatica’ and ‘Black Beauty’ (Figure 6B,C). In addition, drought stress had little effect on GSH and GSSG.

Furthermore, combined and individual stresses reduced the GSH/GSSG ratio in all eggplant cultivars. Combined stresses decreased the GSH/GSSG ratio by 57.5%, 58.7%, 59.25%, and 60% and in ‘Adriatica’, ‘Galine’, ‘Black Beauty’, and ‘Bonica’, respectively, in comparison to control plants; however, heat stress alone decreased the GSH/GSSG ratio by 34.3%, 37.1%, 44.3%, and 46% in ‘Adriatica’, ‘Black Beauty’, ‘Galine’, and ‘Bonica’, respectively, when compared to control. Moreover, drought stress alone declined the GSH/GSSG ratio by 27.7%, 20.1%, 13.5%, and 7.5% in ‘Black Beauty’, ‘Adriatica’, ‘Bonica’, and ‘Galine’, respectively, in comparison to control plants (Figure 6B,C).

### 2.5. Drought-, Heat-, and Combined Drought and Heat-Induced Changes in Hormones: Abscisic Acid (ABA), Salicylic Acid (SA), and Jasmonic Acid (JA)

All applied stresses significantly increased ABA, SA, and JA, but only in ‘Bonica’ and ‘Galine’.

**Figure 7:**
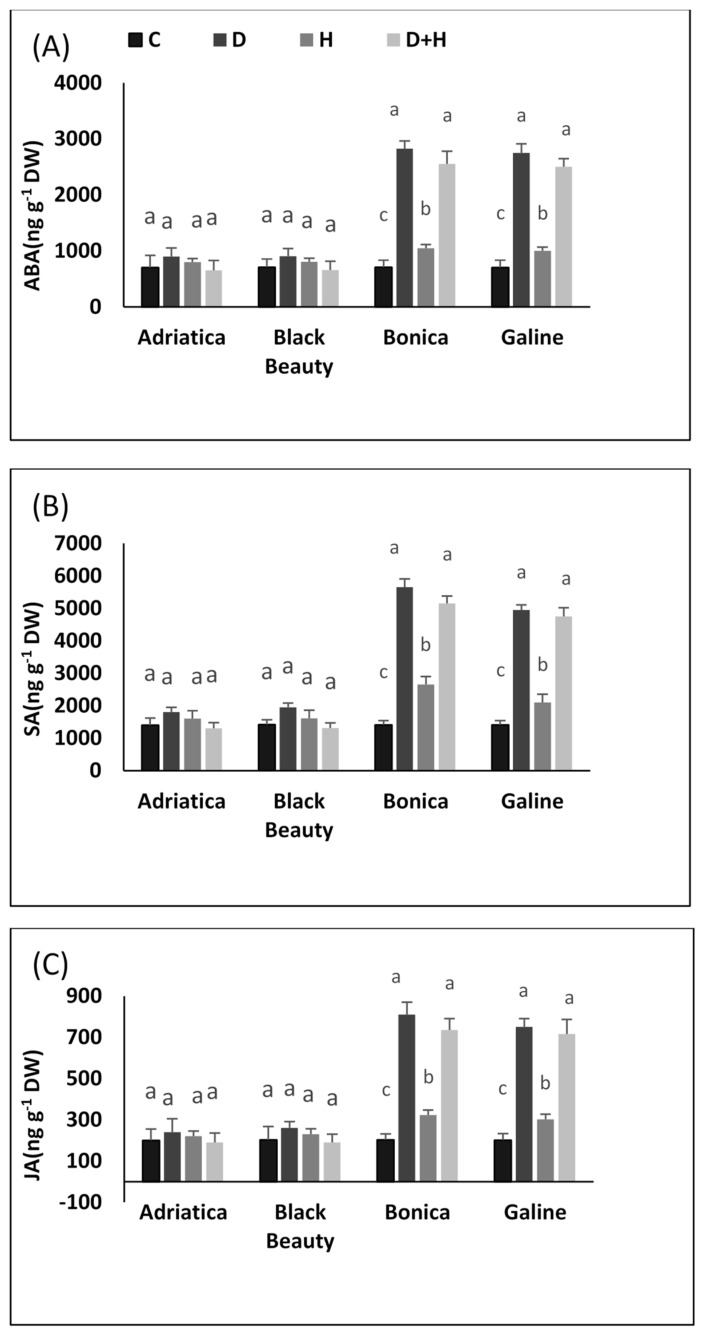
Changes in (**A**): abscisic acid (ABA), (**B**) salicylic acid (SA), and (JA) jasmonic acid (**C**) of four eggplant cultivars under individual and associated stresses. Data presented are the means ± SE (*n* = 5). Significant dissimilarities are shown by different lower-case letters between treatments (*p* ≤ 0.05) based on Tukey’s test. (C, control; H, heat stress; D, drought stress; H + D, heat + drought stresses).

Drought stress alone rose ABA by 22.2%, 22.28%, 291.1%, and 300.2%, in ‘Adriatica’, ‘Black Beauty’, ‘Galine’, and ‘Bonica’, respectively, in comparison to control plants (Figure 7A). Moreover, individual heat stress increased SA by 14.28%, 14.3%, 48.7%, and 42.5% in ‘Adriatica’, ‘Black Beauty’, ‘Bonica’, and ‘Galine’, respectively, compared to control plants (Figure 7B). Added to that, while combined stresses boosted JA by 261.2% and 256%, respectively, in ‘Bonica’ and ‘Galine’, it slightly declined the same parameter in ‘Adriatica’ and ‘Black Beauty’ (Figure 7C).

### 2.6. Drought-, Heat-, and Combined Drought and Heat-Induced Changes in Nutrient Uptake

**Figure 8:**
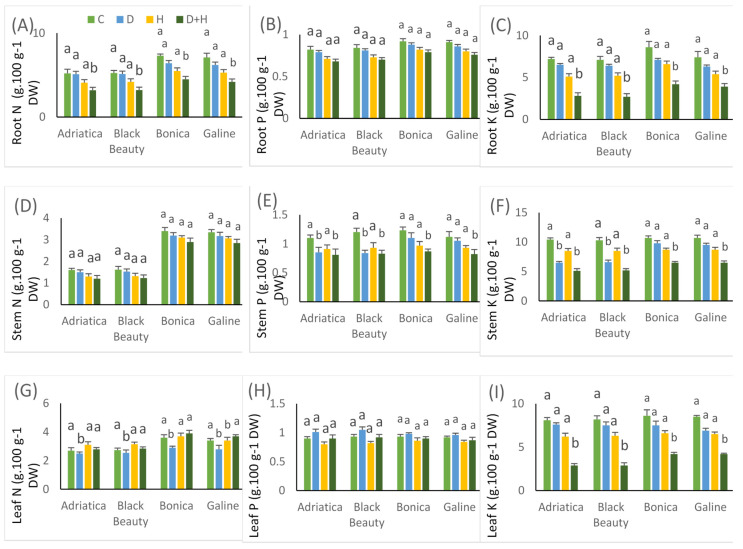
Changes in Nutrient contents in roots: (**A**) nitrogen, (**B**) phosphorus, and (**C**) potassium; in stems (**D**) nitrogen, (**E**) phosphorus, and (**F**) potassium, and in leaves (**G**) nitrogen, (**H**) phosphorus, and (**I**) potassium of four eggplant cultivars subjected to drought, heat, and drought + heat stresses. Data presented are the means ± SE (*n* = 5). Significant dissimilarities are shown by different lower-case letters between treatments (*p* ≤ 0.05) based on Tukey’s test. (C, control; H, heat stress; D, drought stress; H + D, heat + drought stresses).

Compared to control, heat stress hardly affected nitrogen (*N*) content in roots, leaves, and stems of all eggplant cultivars, while individual drought stress significantly reduced the leaf N content in all eggplant varieties (Figure 8). Under individual drought stress, the N content in roots and stems of all eggplant cultivars showed no significant difference when compared to control treatment. Under combined stresses, N content in roots, leaves, and stems of eggplant varieties were in the order of root N > leaves N > stem N. The imposition of drought or heat separately did not affect the content of the root and leaf phosphorus (P) and potassium (K) in all eggplant cultivars, compared to control. However, drought stress alone significantly decreased the P and K content in stems of ‘Adriatica’ and ‘Black Beauty’ (Figure 8). For all eggplant cultivars, the impact of drought and heat stresses on N, P, and K absorption was greater. Under combined drought and heat stresses, the contents of N and K in roots, P and K in stems, and K in leaves decreased significantly in all studied eggplant varieties (Figure 8). Furthermore, for the four eggplants varieties, the P and K contents of the stems and leaves were higher than that of the roots. However, the N, P, and K contents in ‘Adriatica’ and ‘Black Beauty’ roots, stems, and leaves were lower than those of ‘Bonica’ and ‘Galine’, indicating a reduced potential accumulation of nutrients under stressful conditions (Figure 8).

### 2.7. Drought-, Heat-, and Combined Drought and Heat-Induced Changes in ACC Deaminase-Producing Bacteria Activity

#### 2.7.1. Quantitative Estimation of ACC Deaminase Activity

**Figure 9:**
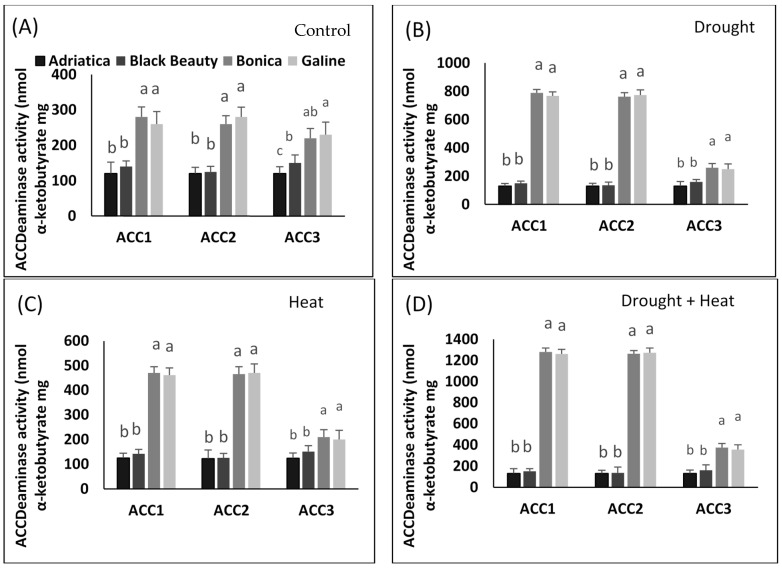
ACC deaminase activity (nmol α-ketobutyrate mg protein^−1^ h^−1^) determined by individual and combined stresses of four isolates from four eggplant cultivars’ roots: (**A**) (Control), (**B**) (Drought), (**C**) (Heat), and (**D**) (Drought + Heat). Values are means of five repetitions ± SE (*n* = 5). Significant dissimilarities are shown by different lower-case letters between treatments (*p* ≤ 0.05) based on Tukey’s test.

The ACC deaminase activity of the studied isolates showed variation, ranging from 450 to 1300 nmol α-ketobutyrate per mg of cellular protein per hour under different types of stress (Figure 9A–D). ‘Bonica’ and ‘Galine’ exhibited higher ACC deaminase activity in all bacterial strains compared to ‘Adriatica’ and ‘Black Beauty’ under all stresses (Figure 9A–D). The most intensive ACC deaminase activity was observed in bacterial strains ‘ACC1’ and ‘ACC2’ in control (varying from 260 to 280 nmol α-ketobutyrate mg protein^−1^ h^−1^), in drought stress (varying from 760 to 790 nmol α-ketobutyrate mg protein^−1^ h^−1^), in heat stress (varying from 460 to 470 nmol α-ketobutyrate mg protein^−1^ h^−1^), and in combined stresses (varying from 1260 to 1300 nmol α-ketobutyrate mg protein^−1^ h^−1^) (Figure 9A–D). The highest enzymatic activity of ACC deaminase was exhibited by ‘ACC1’ and ‘ACC2’.

#### 2.7.2. Estimation of Produced Indole Acetic Acid

The production of IAA was significantly higher in ‘Bonica’ and ‘Galine’ than in ‘Adriatica’ and ‘Black Beauty’ in all stress types for all isolates.

**Figure 10:**
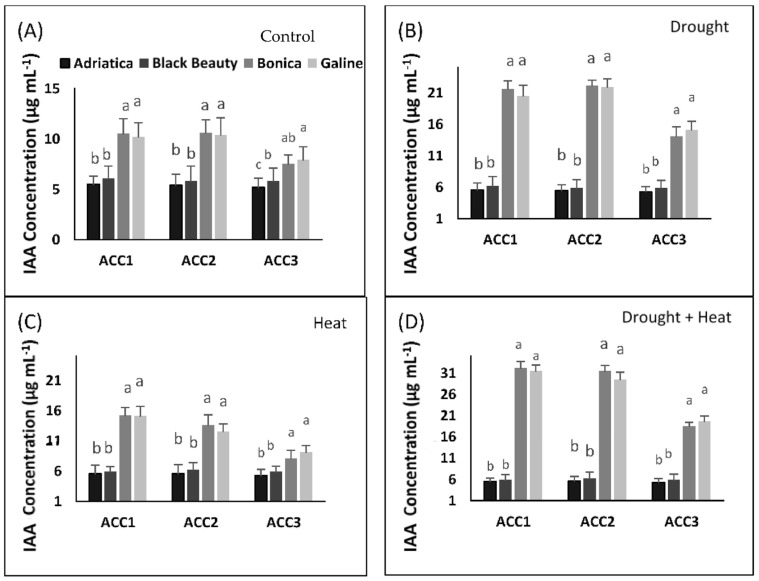
Estimation of IAA production (nmol α-ketobutyrate mg protein^−1^ h^−1^) in response to individual and combined stresses of four isolates from four eggplant cultivars’ roots: (**A**) (Control), (**B**) (Drought), (**C**) (Heat), and (**D**) (Drought + Heat). The values are means of five repetitions ± SE (*n* = 5). Significant dissimilarities are shown by different lower-case letters between treatments (*p* < 0.05) based on Tukey’s HSD test.

The most enhanced IAA accumulation was observed for ‘Bonica’ and ‘Galine’ cultivars by ACC2 for individual drought stress (ranging from 21.9 µg/mL to 22.1 µg/mL) and by ACC1 for combined drought and heat stresses (varying from 31.5 µg/mL to 32.2 µg/mL) (Figure 10A–D).

## 3. Discussion

The onset of single or combined drought and heat stresses is deemed as the most severe abiotic stress, drastically constraining development and production of several crops. Drought and heat stresses deeply alter many vulnerable crop plants’ features such as morphology, growth, and yield [3,14,40]. In the current study, drought and heat stresses decreased plant height and biomass, which significantly reduced yield parameters such as fruit number and fruit weight in all eggplant varieties. However, the effect of combined stresses was sharper than the effect of each stress individually (Table 1 and Table 2). In addition, the impact of all stress treatments was more drastic in ‘Adriatica’ and ‘Black Beauty’ than in Bonica’ and ‘Galine’. Under stressful environments, plants have a tendency to keep their physiological equilibrium via higher RWC, specifically when transpiration rate is elevated. RWC is considered a useful indicator in assessing plant drought tolerance [41,42]. It is worth noting that according to protoplasmic permeability, plants provided with higher RWC are considered more tolerant to drought stress. In the present experiment, ‘Adriatica’ and ‘Black Beauty’ exhibited significant decreases in their RWC, suggesting their susceptibility towards stress (Table 1). However, ‘Bonica’ and ‘Galine’ succeeded in maintaining steady RWC, suggesting their tolerance towards stress. This agrees with previous studies [22,42,43] stating that RWC reflects the plant’s physiological balance and could be considered as an indicator of stress tolerance or susceptibility. Under stress conditions, plant chlorophyll pigments contribute to several metabolic pathways. Chl fluorescence measurements are considered as an early noninvasive tool to evaluate the stress-induced damage in plants. Chlorophyll decline deeply alters plant production and decreases growth and development [42,44,45]. In the present experiment, while ‘Adriatica’ and ‘Black Beauty’ showed a significant reduction in different plant pigments, Bonica’ and ‘Galine’ were not affected under combined and individual stresses. A close relation to the chloroplast impairment could explain this (Table 3). Our results are in coherence with previous studies [24,42,46] conducted under combined stresses showing that chlorophyll measurements are considered a feasible tool to assess the impact of stressful environments on plant. Because of their antioxidant potential, carotenoids preclude photo-oxidation of chlorophyll. Based on their protective potential, carotenoids seem to be more subject to disruption under various stress conditions, which contributes to cellular damage in addition to pigment impairment [47].

Under abiotic stress conditions, chlorophyll fluorescence is considered as an early nondestructive indicator to assess the tolerance or the susceptibly of plants [42,48,49]. To assess the direct impact of the individual and combined stresses on PSII photochemistry, Chl *a* fluorescence was evaluated in four eggplant cultivars. The negative impact of drought stress on the photosynthetic machinery and electron transport chain could be due to a decrease in electron transport or the inhibition of the PSII acceptor side in all cultivars. However, the individual or combined heat and drought-induced changes in chlorophyll fluorescence parameters were more serious in ‘Adriatica’ and ‘Black Beauty’ than in Bonica’ and ‘Galine’.

Drought stress significantly reduced F_V_/F_m_ (Figure 1A) and increased NPQ (Figure 1D) in all eggplant cultivars. The decrease in F_V_/F_m_, as a measure of PSII function, and the rise of energy dissipation as heat and fluorescence could be generated by the buildup of passive PSII reaction centers and the decline of quantum yield of PSII photochemistry in plants [42,50]. Chlorophyll fluorescence measurements showed that drought stress hampered the effective electron transfer rate to PSII reaction centers, mainly caused by the impairment of energy absorption, electron transport, and loss per cross section, which contributes to the decrease in the photosynthetic effectiveness of PSII [42,50,51].

Drought stress progressively reduced PSII electron transport and rose non-photochemical quenching in wheat, which approve the function of a substitute sink (PSII or PSI) and cyclic electron flux for photoprotection of PSII and PSI, thus producing ATP necessary to overcome the effects of drought stress [42,52]. Further, the drought stress moderately reduced absorption and electron transport efficiency in addition to the number of active reaction centers in crop plants [42,53]. According to [54], drought and heat stresses hardly affect F_v_/F_m_ and primary photochemical activities of PSII. In our experiment, under single or combined stresses, F_v_/F_m_ declined impressively, whereas the reduction was more pronounced in ‘Adriatica’ and ‘Black Beauty’ than in ‘Bonica’ and ‘Galine’ (Figure 1). Moreover, the negative impact of combined stresses was sharper than individual stresses. Our results are in coherence with previous studies reported in [42,55,56]. In addition, under all types of stress, q_p_ and ΦPSII decreased, while NPQ rose in all eggplant varieties. However, the stress-induced change in q_p_ and ΦPSII was more drastic in ‘Adriatica’ and ‘Black Beauty’ than in ’Bonica’ and ‘Galine’ (Figure 2III,IV). Our results confirm that drought stress affects CO_2_ availability mainly induced by stomata closure. As already emphasized [57], under combined stresses, the substantial decrease in F_v_/F_m_ could be attributed to a serious disruption in PSII. Drought stress contributes to a photo-inhibition, which is mainly counteracted by increasing heat dissipation via NPQ. The inhibition of electron transport, at the PSII acceptor, drastically reduced F_v_/F_m_ [2,42]. Similarly, it was reported that serious PSII damage was closely linked to both combined and individual heat and drought stresses [58]. Combined heat and drought stresses induced a harsher decrease in photochemical efficiency of PSII [59].

Photosynthesis is known to be the most vulnerable physiological feature to drought and heat stresses. As already pointed out [60], drought stress generates stomatal closure and, consequently, impedes chloroplast CO_2_ supply. Furthermore, it was previously stated that under heat stress, non-stomatal processes such as Rubisco activity and electron transport efficiency are deeply linked to photosynthetic machinery inhibition [61].

In the current study, individual and combined stresses significantly reduced stomatal conductance, transpiration rate, CO_2_ assimilation, and photosynthetic activity. However, a greater harmful impact was observed under combined stress. Moreover, the negative effect of individual and combined stresses was more severe in ‘Adriatica’ and ‘Black Beauty’ than in ‘Bonica’ and ‘Galine’. Our results confirm that plant adaptation to combined drought and heat stresses is closely related to their ability of keeping higher photosynthetic efficiency. While they are coexisting, both heat and drought stresses compete in accelerating photosynthetic apparatus damage and the impairment of all its processes [42].

Drought and heat stresses, alone or combined, differently affected crops. Drought stress altered the water status (leaf water potential and stomatal conductance), the maximum quantum yield of PSII (F_v_/F_m_), and the effective quantum yield of PSII (ΦPSII); however, an increase in the temperature leads to higher photosynthetic rate. In addition, as already described [62], the negative effect on plant physiological traits was more accentuated under combined elevated temperature and drought. Under individual and combined stresses on tobacco [63] and tomatoes [42], similar physiological effects were discovered.

In the present study, individual and combined stresses reduced transpiration rate (E), CO_2_ assimilation rate (A), stomatal conductance (g_s_), and net photosynthesis (P_n_), which is in agreement with several other previous reports [2,24,42,64] (Figure 2I–IV).

It was highlighted that, under drought stress alone, the substantial decrease in plant productivity is closely related to the decline in photosynthetic machinery efficiency under drought stress [65]. According to [60], the impediment of photosynthesis under drought stress is mainly induced by stomatal closure and assimilation transport limitation. In opposition to this, in [42,66,67], it was stated that under a heat stress condition, P_n_ is hampered via photosynthetic biochemical processes. The impediment of P_n_ induced by a decrease in gs is more aggravated under combined drought and heat stresses than under individual drought stress. High osmotic stress generates a decrease in PSII activity, reduction in the effective quantum yield of PSII, and D1 protein damage, thus contributing to the deactivation of the PSII reaction center [42,68]. It was pointed out that individual heat stress hardly affected the photosynthetic rate of tobacco plants [69]. In contrast, drought stress alone and combined drought and stresses lowered the photosynthetic rate by 80%. Under drought and combined stresses, *Cicer arietinum* exhibited higher RuBisCO activity and lower enzymatic activities [70]. Under combined stresses, a strong accumulation of ROS caused a significant decline in photochemical effectiveness of photosystem II (PSII) in *Festuca arundinacea* [59]. Moreover, combined stresses generated a substantial decrease in the photosynthetic rate in *Populus yunnanensis* [71]. However, high photorespiration induced by individual heat stress is considered a principal factor in decreasing Rubisco activity, PSII, and photosynthesis rate [42,72,73,74].

Chl fluorescence could be used as a nondestructive indicator to assess the impact of various abiotic stress on plants such as drought [42], NaCl [75], temperature [76], and ultraviolet radiation [77]. As already emphasized [49,78], the evaluation of photochemical activity through chlorophyll fluorescence provides information on the severity of the studied stresses.

The imposition of abiotic stresses such as drought, heat, and various other types of stress engenders high buildup of ROS (such as superoxide (O_2_^−^), singlet oxygen (O_2_), hydrogen peroxide (H_2_O_2_), and hydroxyl radicals (OH^−^)), which creates high oxidative stress, thus contributing to the impediment of plant metabolism [79,80,81].

Under stress conditions, the continuous ROS accumulation in plant cells, cellular damage components (DNA, membrane lipids, and proteins) affect the integrity and functionality of cell membranes and lead to growth cessation [82,83,84]. In the current experiment, under individual drought, heat, and combined stresses, all eggplant cultivars exhibited elevated H_2_O_2_ and MDA contents, indicating that the extent of oxidative harm is closely linked to the level of predisposition of eggplant varieties to associated drought and heat stresses [2,42].

The subjection of eggplant cultivars to individual and combined stresses increases the buildup of ROS, which in turn damages cell membrane lipids, thus producing MDA, which measures the membrane lipid peroxidation and the extent of oxidation damage under stress [84,85]. Both ‘Adriatica’ and ‘Black Beauty’ had a relatively high level of MDA produced due to peroxidation of membrane lipids under different types of stress. The lower relative increase in MDA exhibited by ‘Bonica’ and ‘Galine’ suggested a lower stress effect (Figure 3E), which should evoke less oxidative damage in these cultivars.

Eggplant cultivars subjected to drought, heat, or combined drought and heat stresses revealed an increase in EL. However, this increase was sharper in ‘Adriatica’ and ‘Black Beauty’ than in ‘Bonica’ and ‘Galine’ (Figure 3F), thus showing a higher lipid peroxidation of membranes for ‘Adriatica’ and ‘Black Beauty’ under individual and combined stresses [85]. Furthermore, elevated membrane lipid peroxidation might be associated with higher ROS species, since more intensive abiotic stress is closely related to higher lipid peroxidation and membrane deterioration [25,84]. Our results are in discordance with previous findings reported for tomato [86] and eucalypts [87], showing that the combinations of drought and heat generate higher protective defense systems against more negative effects of individual drought stress.

Proline is widely known by its key role in plant osmoprotection under stress conditions, thereby modulating their tolerance [42,88,89]. The increased buildup of proline is a common phenomenon observed in plants in stressful environments. In the present study, the assessment of proline levels showed that heat stress accumulated lower levels in comparison to drought and combined stresses (Figure 3C). Moreover, although proline rose under all stress types in all cultivars, the highest relative increment was recorded in ‘Adriatica’ and ‘Black Beauty’, while a lower increase was observed in ‘Bonica’ and ‘Galine’. It was pointed out that proline accumulation was substituted by sucrose accumulation in order to reduce proline toxicity effects in cells [90,91]. In addition, drought and combined stresses generated higher accumulation of proline. This is in agreement with previous work [42,92], evincing that high proline content is combined with plant osmoprotection to overcome associated drought and heat stresses. Under abiotic stress, the catabolic pathway of proline, blocking its oxidation and, thus, contributing to high proline accumulation, can be upregulated. Our findings support various recent reports suggesting that plant proline buildup associated with stressful environments [42,93,94] is deeply linked to its potential in cellular component stabilization, free radical scavenging, and cellular redox buffering [95,96].

RWC could be used as an indicator of drought stress tolerance [41]. Under abiotic stress, plants try to keep their physiological optimum via maintaining water status equilibrium by keeping better RWC levels, specifically while transpiration rate is high. Depending on protoplasmic permeability, plants showing higher RWC are considered drought tolerant. In the current study, while exposed to different types of stresses, all eggplant cultivars showed a decreasing trend. However, ‘Adriatica’ and ‘Black Beauty’ displayed a more drastic decrease than ‘Bonica’ and ‘Galine’, proving their susceptibility to stress (Figure 3A). This in agreement with previous work that has been performed in pea plants under drought stress [43]. The capacity of keeping higher RWC is believed to be a useful indicator of combined stress tolerance in tomato [2]. In addition, higher RWC, combined with other parameters, significantly lowers evaporation, maintains membrane stability, and improves drought tolerance [97].

It is widely known that under abiotic stress, plant chlorophyll pigment plays a key role in numerous metabolic activities. Chlorophyll evaluation is considered a useful tool in assessing environmental stresses’ impacts on plants. Chlorophyll damage is closely related to plant productivity decline, growth reduction, and low stress tolerance [44,45].

In the current experiment, under individual and combined drought and heat stresses, all eggplant cultivars exhibited a decreasing trend in plant pigments; however, the decline was significant only in ‘Adriatica’ and ‘Black Beauty’. This could be explained by the extent of chloroplast impairment depending on the eggplant variety. The decrease in chlorophyll content has been associated with combined stresses in many species such as maize [24], ryegrass [46], and tomato [42].

Carotenoids play a crucial role in photo-oxidation inhibition using their effective antioxidant potential [47]. However, because of their protective capacity, carotenoids are more subject to impairment under stressful conditions, which contributes to cellular damage and pigment impediment.

With conditions of abiotic stress, the endogenous production of ROS is activated and accelerated. The oxidative stress, induced by ROS excessive accumulation, damages cellular metabolism and hampers intracellular communication modulating the capacity of plants to counteract stressful conditions via an effective antioxidant system [75,84,98]. Under stressful environments, plants are provided with an efficient protective system including SOD (superoxide dismutase), which is believed to be the primary defensive reaction against oxidative stress damage.

Based on its enzymatic and non-enzymatic antioxidant potential, ascorbate glutathione is active in different cellular parts, thus effectively contributing to the scavenging of ROS. The AsA–GSH pathway encompasses four enzymatic parts (APX, MDHAR, DHAR, and GR) and two antioxidants [42,99].

While being reduced to water via APX, H_2_O_2_ generates an unsteady component MDHA which, after dismutation, produces AsA and DHA, then a reduction by DHAR yields GSSG from GSH. The production of NADPH by photosynthesis is necessary for GR in the regeneration of GSH using oxidized GSSG [99,100]. The increasing trend of CAT and SOD under both individual and combined stresses exhibited by all eggplant cultivars in the present research has been previously recorded in several reports [2,24,42]. SOD had a major role in converting superoxide anions (O_2_ ^−^) into O_2_ and H_2_O_2_. CAT contributes efficiently to the conversion of H_2_O_2_, which reached a toxic level, into H_2_O and oxygen [101]. Under combined stresses, the elimination of H_2_O_2_ through APX and CAT is believed to be an effective scavenging system, improving plant abiotic stress tolerance [102]. In the present experiment, under individual and combined heat and drought stresses, all eggplant varieties showed a significant enhancement in APX activity, thus indicating the presence of active defensive machinery against the studied stresses; however, the increase displayed by APX was more pronounced in ‘Bonica’ and ‘Galine’ than in ‘Adriatica’ and ‘Black Beauty’, proving their better tolerance to stress (Figure 5B). Our findings are consistent with previous reports [42,103] showing that the effectiveness of antioxidant enzyme machinery is correlated with higher plant tolerance under stress conditions. Under more intensified temperature, the H_2_O_2_ production levels exceeded the scavenging capacities of the plants, while CAT activity is not launched, thereby engendering oxidative stress impairment. Moreover, as already emphasized, the dismutation of H_2_O_2_ is achieved by APX, which utilizes AsA as an electron donor [104]. In the current study, under all studied stresses, DHAR and MDHAR activities and the GSH/GSSG ratio significantly decreased to varying degrees in all eggplant cultivars. The DHAR and MDHAR enzymes’ initiation is involved in GSH and AsA synthesis pathways, thus providing GSSG to GR and AsA to APX [105]. It was highlighted that the enhanced activity of MDHAR and DHAR indicates that, following the reduction of H_2_O_2_ to H_2_O via APX, the generated ROS were immediately transferred to AsA through MDHAR or via spontaneous various pathways [106].

It was previously reported that the conversion of GSSG to GSH contributes effectively to maintaining a better GSH/GSSG ratio [107]. Under stressful environments, increasing GR activity improve the plant’s ability in keeping a high NADP^+^ pool and protecting photosynthetic machinery against photo-oxidation. A well-balanced GSH/GSSG ratio is crucial for maintaining optimal cell development [42,108]. Under abiotic stress conditions, plants are provided by an active antioxidant enzyme system that includes ascorbate and glutathione, which are effective in avoiding cell membrane oxidation through their redox buffering potential [99]. In addition, AsA and GSH play a key role in supplying APX and GPX by electrons [109].

Under combined drought and heat stresses, all eggplant varieties displayed an increasing trend in AsA, DHA, and the AsA/DHA ratio, reflecting the high magnitude of stress (Figure 6C,D and Figure 7A). The preservation of an optimal GSH/ GSSG ratio is crucially needed for a cell redox control that implies an accurate regulation of the GSH cycle. In this context, at the expenditure of NADPH, GR activity is believed to be an effective user of GSH [104]. Under all types of stress, eggplant cultivars showed a declining trend in the GSH/GSSG ratio, suggesting a disruption in GSH recycling. As stated previously by [104], the keeping-up of the GSSG balance is closely related to the extent of ROS buildup. This is consistent with our findings, while MDA exhibited a significant increment in all eggplant varieties under combined stresses (Figure 3E).

Furthermore, under combined stresses, the recorded GR activity (Figure 6B) seems to be insufficient in maintaining an optimal GSH/GSSH ratio, thus contributing to the inhibition of the ROS scavenging system [42,110].

In the present study, under heat stress, the increasing tendency exhibited in SA and JA was significant only in ‘Bonica’ and ‘Galine’, which could be explained by their superior ability to use the hormonal pathway more effectively (SA and JA), which is deeply involved in signaling and tolerating abiotic stress tolerance [111,112,113].

JA and ethylene contribute effectively to enhancing defensive processes against necrotrophic pathogens and herbivorous insects [114]. Consequently, this result confirms the effect of abiotic stress on biotic interactions, thus indicating that these abiotic stresses can deeply hamper plants’ defense capacities to overcome other biotic threats.

It was highlighted that the efficiency of absorption, buildup, and delivery of N, P, and K to plant organs deeply influence plant growth and productivity performance [115]. Temperature variations may induce substantial changes in plant nutrient uptake pathways, whereas the magnitude of these changes is mainly dependent on the plant organ and the physiological activity [116]. In the current study, individual heat stress hardly affected the N, P, and K content in all plant organs of all eggplant cultivars (Table 3). Under elevated temperature on Douglas fir (Pseudotsuga menziesii), no significant effect was discovered in plant N uptake, whereas root and woody tissue N content declined compared to control [117]. Individual drought stress significantly reduced the leaf N content in all cultivars and stem P and stem K content in ‘Bonica’ and ‘Galine’ when compared to the control (Table 3). It was pointed out that drought stress severely affects the macronutrient (N, P, and K) absorption and distribution in numerous crops [118,119].

It is worth noting that the negative impact of combined drought and heat stresses was more accentuated concerning nutrient uptake than the individual impact of these stresses. In comparison to control, combined drought and heat stresses drastically declined content of N and K in roots, P and K in stems, and K in leaves of all eggplant cultivars (Figure 8). This could be explained by the decrease in the presence, absorption, and delivery of these nutrient elements under abiotic stress. As already emphasized, individual drought and heat stresses, especially in combination, strongly hamper nutrient cycling, absorption, availability, and use in plants through the impediment of several physiological activities [3,14].

## 4. Materials and Methods

### 4.1. Experimental Designs and Stress Treatments

Seeds of four commercial eggplant (*S. melongena* L.) cultivars, i.e., two open-pollinated (‘Adriatica’ and ‘Black Beauty’) and two F1 hybrids (‘Bonica’ and ‘Galine’), were first treated with 70% alcohol for a few seconds and then rinsed with distilled water. Later, the seeds were sterilized in a 5% HazTab solution (1,3,5 dichloro-triazine-trionedihydrate-dichlorosodium) and 0.02% Dreft (5–15% non-ionic surfactants, 15–30% anionic surfactants) for twenty minutes and, subsequently, with a solution of mercuric chloride (0.5%) for 10 min. The seeds were then rinsed three times with distilled water, and sown into 2 L plastic pots (3 seeds/pot) containing a peat-based medium. One plant per pot was kept after the thinning of seedlings. Plants were watered every two days with full-strength Hoagland nutrient solution (250 mL) over 15 days. Plants were placed in a glasshouse (at a temperature of 25 °C, RH of 70%, and photon flux density of 340 μmol m^−2^ s^−1^) (36°50′ N, 10°11′ E) at the Department of Plant Physiology and Biotechnology, National Institute of Agronomic Research, Tunisia. Four experimental groups of plants were implemented. In the first group (the control), plants were grown at 25 °C and irrigated every day with full-strength Hoagland solution.

In the second group (drought stress), plants were subjected to drought stress, irrigated with full-strength Hoagland solution over one week, then the plants were maintained without water over ten days in order to reduce their relative water content it reached 60%. In the third group (heat stress), plants were subjected to a temperature of 45 °C over one week. Finally, in the fourth group (combined drought and heat stresses), plants were first exposed to drought stress, then subjected to heat stress treatment (temperature of 45 °C over 24 h). At the end of the experiment, the uppermost fully expanded young leaves were collected and maintained in liquid nitrogen for biochemical and antioxidant analyses.

### 4.2. Growth and Yield Parameters Assessment

By the end of each stress application, twelve plants (3 plants per block) were harvested randomly for every stress type. The height and fresh weight (FW) were measured. Dry weight (DW) was determined after 48 h of drying at 60 °C in a forced-draft oven. The harvest of fruits began 40 days after flowering, and they were counted and weighed.

### 4.3. Relative Water Content and H_2_O_2_ Level

At the end of the experiment, topmost fully grown leaf samples were collected and utilized for the determination of leaf RWC, which was achieved following the previously published methodology [120]. The leaf fresh weight (FW), turgid weight (TW), and dry weight (DW) were measured and RWC was calculated as follows:

RWC (%) = [(FW − DW)/(TW − DW)] × 100

Hydrogen peroxide content was assessed according to [121]. In short, the homogenization of frozen eggplant leaves (0.25 g) was achieved in an ice bath using 1 mL of 0.1% (*w*:*v*) TCA and a centrifugation of the homogenate was performed at 12,000× *g* over 15 min at 4 °C.

The aliquots of 100 μL from each tube were placed in 96-well plates which were supplemented by 50 μL of 10 mM potassium phosphate buffer (pH 7.0) and 100 μL of 1 M KI. The standard curve was performed using commercial H_2_O_2_. After being vortexed, the incubation of the plate was conducted over 30 min at room temperature and the absorbances were recorded at 390 nm in a microplate reader. H_2_O_2_ content was measured based on the standard curve.

### 4.4. Pigments and Metabolites Analysis

Chlorophyll a, b, and carotenoids content was assessed as described in the literature [122]. The extraction of pigments was achieved using cold acetone:50 mM Tris buffer, pH 7.8 (80:20) (*v*/*v*). After centrifugation, supernatant absorbance was recorded at 470, 537, 647, and 663 nm (Thermo Fisher Scientific Spectrophotometer, Genesys 10-uv S, Light Machinery, Ottawa, ON, Canada). By the end, every pigment concentration was estimated as described in the literature [122] and expressed in µg g^−1^ fresh weight (FW).

Lipid peroxidation was assessed by the quantification of malondialdehyde (MDA) according to [75,123] by using 1 g of leaf in 80% ethanol. The quantification of MDA was based on the reaction with thiobarbituric acid (TBA). Then, the absorbance values were read at λ = 440 nm, 532 nm, and 600 nm by a spectrophotometer (InfiniteM200 TECAN Group Ltd., Zürich, Switzerland). Malondialdehyde (MDA) equivalents were determined according to [123].

Proline quantification was achieved according to [124]. Briefly, the extraction of 500 mg of plant tissue was performed using 3% (*w*/*v*) sulfosalicylic acid. Proline content was estimated as µg proline g^−1^ FW.

Starch determination was performed according to [125]. Briefly, the extraction of sugars was achieved using 20 mg fresh weight supplemented by 1 mL of ethanol (80%) at 70 °C for 10 min and further at 45 °C for 3 h, followed by centrifugation at 5000× *g* for 5 min. The rest of the ethanol-insoluble material was washed twice using 80% ethanol, and the pellet was treated with HCl 1M during 2 h at 95 °C for starch hydrolysis. Starch extraction and quantification were carried out following the protocol of [125] and based on the enzymatic reduction in NADP+ (UV-VIS, Biotek Uvikon XL, Santa Clara, CA, USA).

### 4.5. Electrolyte Leakage (EL)

Electrolyte leakage (EL) was evaluated following the protocol of [126]. In short, after 25 days of stress application, three leaf disks (2 mm in diameter) were cut from three chosen and fully washed expanded leaves. After being placed in a glass vessel with 10 mL of sterile distilled water, disks were shaken for 5 h, then the initial EC1 was recorded using an Electrical Conductivity Meter (Beckman, instrument Inc., Cedar Grove, NJ, USA). Then, the EC2 was determined after autoclavation of leaf disks (121 °C over 15 min). By the end, the EL (%) was calculated as follows: EL (%) = (EC1/EC2) × 100.

### 4.6. Gas Exchange Measurements

To assess the effect of individual and combined stresses on photosynthetic parameters, measurements were conducted once a day (between 09:00 h and 12:00 h) on four randomly chosen plants. This was done after a period of 25 days of applying stress. Measurements were carried out on sunny days using a portable photosynthesis system (model LI-6400; Li-Cor Biosciences, Lincoln, NE, USA). Carbon dioxide assimilation (A, µmol CO_2_ m^−2^ s^−1^), stomatal conductance (g_s_, mmol H_2_O m^−2^ s^−1^), net photosynthetic rate (Pn, µmol m^−2^ s^−1^), and transpiration rates (E, mmol H_2_O m^−2^ s^−1^) were determined in the selected plants.

Chl *a* fluorescence was measured in dark- and light-adapted leaves with a portable fluorometer (PAM-2500, Walz, Effeltrich, Germany). After 30 min of dark adaptation, F_v_/F_m_ was estimated as (F_m_ − F_0_)/F_m_, where F_m_ (induced by a short pulse (0.6 s) of saturating light (3450 µmol m^−2^ s^−1^)) and F_0_ were the maximum and minimum fluorescence, respectively [127]. After 4 min of illumination with continuous red, nonsaturating actinic light (447 µmol m^−2^ s^−1^) and saturating pulses every 25 s, the maximum (F_m_’) and the constant-state (F_s_) fluorescence signals were determined in the light-adapted leaves. Then, the actinic light was turned off and the far-red pulse was used to obtain the minimal fluorescence after the PSI excitation (F_0′_). ΦPSII was expressed as (F_m_’ − F_s_)/F_m_’ while q_p_ was assessed as (F_m_’ − F_s_)/(F_m_’ − F_0′_) [128]. Finally, NPQ was calculated as (F_m_ − F_m_’)/F_m_’ [129].

### 4.7. Enzymatic Assays

For protein and enzyme extractions, the sample (0.5 g of leaf) was homogenized with a buffer of potassium phosphate (50 mM, pH 7.8), with 1 mM EDTA-2Na and 7% (*w*/*v*) polyvinylpolypyrrolidone (PVPP). The extraction was done at a temperature of 4 °C. The centrifugation of the homogenates was performed at 4 °C over 15 min at 13,000× *g*, and the enzyme activity was assayed utilizing the supernatants. Protein was investigated as previously described [130], using bovine serum albumin as a standard.

Superoxide dismutase activity (SOD; EC1.15.1.1) was quantified by the capacity of the enzyme to impede the light-dependent reduction of nitro blue tetrazolium chloride (NBT). The absorbance of the mixture was recorded at 560 nm and the quantity of enzymes necessary to inhibit the photoreduction rate of NBT by 50% was estimated as one unit of SOD activity, expressed as enzyme units (EU) per mg of protein.

According to [131], we assayed catalase (CAT) activity (EC 1.11.1.6), which was examined by measuring the level of reduction of H_2_O_2_ (ε = 2.3 mM^−1^ cm^−1^) at 240 nm. This activity was determined in a reaction mixture containing 1900 µL of potassium phosphate buffer (50 mM, pH 7.0, not containing EDTA), 100 µL sample, and 1000 µL H_2_O_2_ (30 mM). CAT activity was expressed as EU mg^−1^ of protein.

Ascorbate peroxidase (APX) activity (EC 1.11.1.11) was assessed according to previously published methodology [131]. The reaction mixture contained 50 mM of potassium phosphate buffer (pH 7.0), 4.4 µL ascorbate (1 mM), and 10 µL EDTA-2Na (0.5 M). Adding H_2_O_2_ started the reaction, and ascorbate oxidation was recorded at 290 nm for 3 min. Activity was determined using the extinction coefficient, e = 2.8 mM^−1^ cm^−1^. Each sample was assayed in three replications. Results were expressed in EU mg^−1^ of protein.

According to published methodology [132], the glutathione reductase (GR; EC 1.6.4.2) activity was quantified by evaluating the decrease in absorbance, at 340 nm, over 3 min, of the reaction mixture with GSSG and NADPH. Results were expressed as EU mg^−1^ of protein.

Monodehydroascorbate reductase (MDHAR; EC1.6.5.4) activity was determined according to [133]. The change in absorbance of both reaction mixtures was assayed spectrophotometrically at 340 nm over 3 min. Results were expressed as EU mg^−1^ protein. Dehydroascorbate reductase (DHAR; EC 1.8.5.1) was investigated using the protocol of [134] by recording the absorbance at 265 nm over 3 min.

### 4.8. Non-Enzymatic Antioxidants Assay

Fresh leaf samples (0.5 g) were homogenized in mixture of 3 mL ice-cold metaphosphoric acid (5%) and 1 mM EDTA. Then, the centrifugation of the homogenate was performed over 10 min at 10,000× *g*. With regard to the assay of total and reduced ascorbate, 400 μL of supernatant was placed in two sterilized Eppendorf tubes. Then, 200 μL of 10% TCA was supplemented to every tube and vortexed before centrifugation and 10 μL of NaOH solution was added to it. Afterwards, 200 μL of 150 mM NaH_2_PO_4_ was supplemented to 200 μL of the supernatant, along with 200 μL of water. An amount of 100 μL of 10 mM DDT and 200 μL of buffer were added to another 200 μL of supernatant and mixed perfectly. To every tube, 100 μL of 0.5% N-ethylmaleimide was then supplemented, vortex mixed, and incubated at room temperature for 30 min. Afterwards, every tube was then complemented with 400 μL of 44% H_3_PO_4_, 4 μL of 4% bipyridyl, 200 μL of 3% FeCl_3_, and 400 μL of 10% TCA, vortex mixed, and then the incubation of the samples was performed for 60 min at 33 °C. Finally, the absorbance of the supernatant was utilized for the assay [134] by measuring spectrophotometrically at 525 nm.

Glutathione pool was determined following the method of [134]. The level of GSH and GSSG was estimated from the standard curves calculated starting from fixed amounts of GSH and GSSG.

At 4 °C, a mush of fresh leaf material (0.5 g) was made by homogenizing them in 2 mL of 5% sulphosalicylic acid. After centrifugation for 10 min at 10,000× *g*, an aliquot of 0.5 mL from the resulted supernatant was placed in a sterilized Eppendorf tube, then 40 μL of DTNB and 0.6 mL of reaction buffer were supplemented to the tube. Two minutes later, the absorbance was recorded at 412 nm to estimate GSH concentration. Then, 2 μL of GR and 50 μL of NADPH were supplemented to assess the total glutathione in the sample.

The oxidized glutathione was estimated by subtracting the reduced form from the total glutathione pool. The reaction took place over 30 min at 25 °C. The absorbance variation was measured at 412 nm using the UV–VIS spectrophotometer (Model DU 640, Beckman, Brea, CA, USA). The obtained results were corrected for the absorbance of supernatant and DTNB.

### 4.9. Hormone Analysis

The extraction and quantification of abscisic acid (ABA), jasmonic acid (JA), and salicylic acid (SA) were achieved according to [135]. Fifty milligrams of frozen and dried leaf samples were supplemented with 100 ng of ABAd_6_, 100 ng of Sad_6_, and 100 ng of dihydrojasmonic acid and homogenized with 5 mL of distilled water. The resulting supernatants, following a cold centrifugation, were recovered and had their pH values adjusted to 3 with 30% acetic acid. After being acidified, the water extract was divided twice against 3 mL of diethyl ether. Then, the recovery and vacuum evaporation of organic upper layer were performed using a centrifuge concentrator (SpeedVac, Jouan, Saint Herblain, France). The dry residue was then resuspended in a 10% methanol solution through slight sonication. The obtained solution was transferred via 0.22 µm regenerated cellulose membrane syringe filters (Albet S.A., Barcelona, Spain) and immediately injected into a UPLC system (Acquity SDS, Waters Corp., Milford, MA, United States). Analytes were segregated by inversed-phase (Nucleodur C18, 1.8 µm 50 × 2.0 mm, MachereyNagel, Barcelona, Spain) using a linear gradient of ultrapure water (A) and methanol (B) (both supplemented with 0.01% acetic acid) at a flow rate of 300 µL min^−1^. The gradient followed was: (0–2 min) 90:10 (A:B), (2–6 min) 10:90 (A:B), and (6–7 min) 90:10 (A:B). The quantitative determination of hormones was performed using a Quattro LC triple quadrupole mass spectrometer (Micromass, Manchester, United Kingdom), connected online to the output of the column via an orthogonal Z-spray electrospray ion source. The hormone quantification was achieved after external calibration against the standards.

### 4.10. ACC Deaminase-Producing Bacteria Assay

#### 4.10.1. Collection of Rhizospheric Soil Sample

Root samples of ‘Adriatica’, ‘Black Beauty’, ‘Bonica’, and ‘Galine’ were taken from 6 pots of each eggplant variety in each treatment during April and May 2021. Four plants/cultivars/treatments were selected from every pot and brought to the laboratory in closed plastic bags for further analysis. The roots were isolated from each plant, crushed, and mixed together to form one composite pool of root sample.

#### 4.10.2. Isolation of Rhizobacteria with ACC Deaminase Activity

The isolation of bacteria and the qualitative estimation of ACC deaminase activity were performed following the protocol from [136]. The bacteria were isolated from the root sample by a means of a serial dilution technique in Luria–Bertani (LB) medium. The colonies with different morphology were subjected to an activity screening with ACC deaminase on sterile minimal DF (Dworkin and Foster) media, corrected with 3 mM ACC as the sole nitrogen source [137]. The plates were inoculated and then incubated in a shaking incubator at 200 rpm at 28 °C for 3 days, and the growth was estimated on a daily basis. Three strains had the ability to survive under drought stress of 60% relative water content, heat stress of 45 °C, or a combination of them. These strains were added with 3 mM ACC as a nitrogen source, implying ACC deaminase activity. The ACC deaminase activity of these three isolates, namely ‘ACC1’, ‘ACC2’, and ‘ACC3’, was evaluated for α-ketobutyrate production.

#### 4.10.3. Quantification of ACC Deaminase Activity

The assessment of ACC deaminase activity was carried out according to known protocol [136] investigating the amount of α-ketobutyrate resulting from the cleavage of ACC by ACC deaminase. The ACC deaminase activity was expressed in mmol α-ketobutyrate mg protein^−1^ h^−1^.

#### 4.10.4. Indole Acetic Acid Production by Bacterial Isolates

According to [138], the strains of bacteria were inoculated in LB medium (corrected with 5 mM tryptophan) and then incubated in an orbital shaker (200 rpm) for 7 days at 28 °C. The determination of IAA was performed using the colorimetric method via Salkowski reagent (0.5 M FeCl_3_ + 70% perchloric acid). The red color (denoting the formation of indolic compounds) was measured by UV–vis spectrophotometer at 530 nm [139]. The determination of IAA was done by using a standard curve of pure indole-3-acetic acid (IAA, Hi-media) ranging between 0 and 100 mg mL^−1^.

### 4.11. Statistical Analysis

All analyses were carried out using a completely random design. The significant differences between treatments or varieties were calculated with SPSS Statistics 21 following a one-way analysis of variance (ANOVA). The means’ comparison was done using Tukey’s multiple range test (*p* = 0.05). All statistical analyses were completed employing SPSS 25 (IBM SPSS Statistics).

## 5. Conclusions

Our results indicate that imposition of drought, heat, and drought + heat stresses affect growth, yield components, and various cellular processes in distinct ways, explaining the stress sensitivity/tolerance of the eggplant cultivars. The impact of combined drought and heat stresses on photosynthetic parameters, osmolyte accumulation, enzymatic and non-enzymatic antioxidant machinery, and mineral uptake of eggplant cultivars were more drastic than their single-factor impacts. Excessive ROS accumulation in stressful environments leads to increased oxidative stress and cell membrane peroxidation, impairing photosynthetic machinery and inhibiting development. The ROS scavenging system differed between eggplant cultivars. Overall, the morpho-physiological growth and yield performance of ‘Bonica’ and ‘Galine’ cultivars were superior to those of ‘Adriatica’ and ‘Black Beauty’ cultivars. When compared to ‘Adriatica’ and ‘Black Beauty’, the higher tolerance of ‘Bonica’ and ‘Galine’ to heat and drought stresses was attributed to highly effective antioxidant enzymatic machinery defenses, better osmolyte pilling-up, and preservation of photosynthetic pigments and mineral uptake equilibrium.

Finally, two putative strains, ACC1 and ACC2, were found to possess other growth-promoting properties, such as the production of IAA. In ‘Bonica’ and ‘Galine’, the strains’ efficacy in minimizing combined or separated drought and heat stressors and stimulating plant development was obvious.

## Figures and Tables

**Table 1:** Effect of drought, heat, and combined drought and heat on height **(%)**, fresh weight (FW) (%), and dry weight (DW) (%) of four eggplant cultivars. Results are expressed as percentage of control.

Variety	Treatments	Height (%)	FW (%)	DW (%)
**‘Adriatica’**	C	100 ± 0.0 a	100 ± 0.0 a	100 ± 0.0 a
	D	56.4 ± 6.8 c	56.3 ± 4.5 b	72.4 ± 9.2 b
	H	82.8 ± 5.2 b	68.3 ± 7.3 b	86.6 ± 6.5 b
	H + D	46.9 ± 7.3 d	11.7 ± 4.3 c	30.5 ± 5.4 c
**‘Black Beauty’**	C	100 ± 0.0 a	100 ± 0.0 a	100 ± 0.0 a
	D	59.4 ± 8.2 c	58 ± 5.5 b	79.1 ± 6.7 b
	H	83.4 ± 5.2 b	70.8 ± 4.7 b	92.8 ± 8.1 b
	H + D	47.2 ± 6.3 d	13.7 ± 4.2 c	26.4 ± 4.8 c
**‘Bonica’**	C	100 ± 0.0 a	100 ± 0.0 a	100 ± 0.0 a
	D	78.2 ± 5.3 b	73.4 ± 9.1 c	74 ± 5.8 c
	H	95 ± 7.2 b	91.8 ± 5.2 b	89.7 ± 4.2 b
	H + D	58.9 ± 3.9 c	52.4 ± 6.3 d	53.7 ± 4.7 d
**‘Galine’**	C	100 ± 0.0 a	100 ± 0.0 a	100 ± 0.0 a
	D	78.1 ± 7.1 b	73.4 ± 6.1 c	73.9 ± 3.9 c
	H	94.7 ± 6.4 b	91.8 ± 4.1 b	89.7 ± 4.7 b
	H + D	58.8 ± 5.3 c	52.4 ± 5.2 d	53.7 ± 6.5 d

The values are means (±standard errors) of four repetitions. Different lowercase letters within each treatment (*n* = 4) represent significant differences among cultivars, based on Student’s *t*-test at 5 % level. (C, control; H, heat stress; D, drought stress; H + D, heat + drought stresses).

**Table 2:** Yield attributes of four eggplant cultivars exposed to drought, heat, and drought + heat stresses harvested 40 days after flowering.

Parameter	Treatment	‘Adriatica’	‘Black Beauty’	‘Bonica’	‘Galine’
Fruit number	C	6 ± 1.9 a	6 ± 1.7 a	7 ± 1.6 a	6 ± 0.5 a
	D	2 ± 1.2 b	2 ± 1.3 b	3 ± 1.2 b	3 ± 1.1 b
	H	3 ± 1.5 b	3 ± 1.2 b	5 ± 1.2 b	4 ± 2.3 b
	D + H	1 ± 1.3 c	1 ± 0.9 c	2 ± 1.7 c	2 ± 3.2 c
Fruit weight	C	180 ± 1.2 a	182 ± 1.6 a	210 ± 1.1 a	250 ± 2.8 a
	D	80 ± 3.6 c	79 ± 2.3 c	130 ± 2.2 c	124 ± 3.1 c
	H	150 ± 0.9 b	155 ± 1.9 b	160 ± 2.9 b	180 ± 1.8 b
	D + H	40 ± 3.2 d	38 ± 2.1 d	80 ± 0.8 d	73 ± 2.1 d

The values are means (±standard errors) of four repetitions. Different lowercase letters within each treatment (*n* = 4) represent significant differences among cultivars, based on Student’s *t*-test at 5 % level. (C, control; H, heat stress; D, drought stress; H + D, heat + drought stresses).

**Table 3:** Effect of Drought, heat, and combined drought and heat on chlorophyll a (Chl*a*), chlorophyll b (Chl*b*), Chl*a/b*, total chlorophyll (Chl*a+b*), and carotenoids in leaves of the eggplant cultivars after 30 days of stress application.

cv	Treatments	Chl*a* (µg g^−1^ FW)	Chl*b* (µg g^−1^ FW)	Chl*a/b*	Chl*a+b* (µg g^−1^ FW)	Carotenoids (µg g^−1^ FW)
‘Adriatica’	C	764.9 ± 2.1 a	351.2 ± 4.5 a	2.17 ± 4.5 a	1116.1 ± 2.5 a	269.3 ± 5.9 a
	D	695.8 ± 2.7 b	346.2 ± 2.1 b	2.00 ± 3.3 b	1042.0 ± 2.7 b	237.5 ± 2.5 b
	H	332.5 ± 1.2 c	261.3 ± 2.3 c	1.27 ± 1.3 c	593.8 ± 2.1 c	149.9 ± 2.1 c
	H + D	161.7 ± 2.5 d	160.2 ± 2.3 d	1.01 ± 4.3 d	321.9 ± 3.2 d	113.1 ± 3.2 d
‘Black Beauty’	C	766.5 ± 1.7 a	355.3 ± 3.4 a	2.15 ± 3.4 a	1121.8 ± 3.4 a	274.2 ± 2.5 a
	D	691.8 ± 1.4 b	351.1 ± 3.5 b	1.97 ± 2.2 a	1042.9 ± 3.3 b	242.3 ± 4.3 b
	H	337.5 ± 1.5 c	266.2 ± 1.2 c	1.26 ± 2.1 b	603.7 ± 1.3 c	153.7 ± 1.3 c
	H + D	156.8 ± 2.2 d	164.5 ± 4.1 d	0.95 ± 3.1 c	321.3 ± 3.2 d	118.5 ± 2.3 d
‘Bonica’	C	617.5 ± 2.7a	304.1 ± 1.3 a	2.03 ± 8.6 a	921.6 ± 3.2 a	147.6 ± 2.8 a
	D	515.3 ± 3.2 a	280.2 ± 3.7 a	1.83 ± 6.9 a	795.5 ± 2.3 a	140.8 ± 1.9 a
	H	450.1 ± 3.6 a	250.6 ± 2.6 a	1.79 ± 3.5 a	700.7 ± 5.2 a	124.8 ± 1.7 a
	H + D	405.1 ± 2.5 a	202.5 ± 3.3 a	2.00 ± 2.9 a	607.6 ± 3.6 a	105.7 ± 1.6 a
‘Galine’	C	614.4 ± 3.2 a	298.2 ± 3.1 a	2.06 ± 3.4 a	912.6 ± 4.1 a	142.5 ± 1.3 a
	D	511.2 ± 1.2 a	277.1 ± 1.2 a	1.84 ± 2.1 a	788.3 ± 1.1 a	132.7 ± 2.5 a
	H	445.3 ± 5.2 a	244.5 ± 3.2 a	1.82 ± 7.5 a	689.8 ± 3.3 a	120.2 ± 3.2 a
	H + D	400.1 ± 2.5 a	200.5 ± 3.3 a	1.99 ± 1.3 a	606.6 ± 4.2 a	103.5 ± 2.1 a

Data are means ± SE of four repetitions. Significant differences are shown by different lowercase letters between treatments (*p* = 0.05) based on Tukey’s HSD test. (C, control; H, heat stress; D, drought stress; H + D, heat + drought stresses).

## Data Availability

No new data were created or analyzed in this study. Data sharing is not applicable to this article.
